# Modeling the Metabolic Costs of Heavy Military Backpacking

**DOI:** 10.1249/MSS.0000000000002833

**Published:** 2021-11-29

**Authors:** DAVID P. LOONEY, ELIZABETH M. LAVOIE, SAI V. VANGALA, LUCAS D. HOLDEN, PETER S. FIGUEIREDO, KARL E. FRIEDL, PETER N. FRYKMAN, JASON W. HANCOCK, SCOTT J. MONTAIN, J. LUKE PRYOR, WILLIAM R. SANTEE, ADAM W. POTTER

**Affiliations:** 1 US Army Research Institute of Environmental Medicine (USARIEM), Natick, MA; 2 Oak Ridge Institute for Science and Education (ORISE), Oak Ridge, TN; 3 Center for Research and Education in Special Environments, Department of Exercise and Nutrition Sciences, University at Buffalo, Buffalo, NY

**Keywords:** ENERGY EXPENDITURE, HIKING, METABOLISM, OXYGEN COST

## Abstract

**Introduction:**

Existing predictive equations underestimate the metabolic costs of heavy military load carriage. Metabolic costs are specific to each type of military equipment, and backpack loads often impose the most sustained burden on the dismounted warfighter.

**Purpose:**

This study aimed to develop and validate an equation for estimating metabolic rates during heavy backpacking for the US Army Load Carriage Decision Aid (LCDA), an integrated software mission planning tool.

**Methods:**

Thirty healthy, active military-age adults (3 women, 27 men; age, 25 ± 7 yr; height, 1.74 ± 0.07 m; body mass, 77 ± 15 kg) walked for 6–21 min while carrying backpacks loaded up to 66% body mass at speeds between 0.45 and 1.97 m·s^−1^. A new predictive model, the LCDA backpacking equation, was developed on metabolic rate data calculated from indirect calorimetry. Model estimation performance was evaluated internally by *k*-fold cross-validation and externally against seven historical reference data sets. We tested if the 90% confidence interval of the mean paired difference was within equivalence limits equal to 10% of the measured metabolic rate. Estimation accuracy and level of agreement were also evaluated by the bias and concordance correlation coefficient (CCC), respectively.

**Results:**

Estimates from the LCDA backpacking equation were statistically equivalent (*P* < 0.01) to metabolic rates measured in the current study (bias, −0.01 ± 0.62 W·kg^−1^; CCC, 0.965) and from the seven independent data sets (bias, −0.08 ± 0.59 W·kg^−1^; CCC, 0.926).

**Conclusions:**

The newly derived LCDA backpacking equation provides close estimates of steady-state metabolic energy expenditure during heavy load carriage. These advances enable further optimization of thermal-work strain monitoring, sports nutrition, and hydration strategies.

The US Army Load Carriage Decision Aid (LCDA) is a software mission-planning tool intended to provide accurate predictions of the metabolic cost of warfighter tasks, time to exhaustion, and associated indicators of physiological strain. The LCDA predicts physiological responses of warfighters during dismounted operations using biomedical models developed at the US Army Research Institute of Environmental Medicine (USARIEM) (1,2). Notably, USARIEM developed equations in the 1970s and 1990s that predict the metabolic costs of foot marches with military loads over complex terrain (3–5). However, these earlier equations underestimate the metabolic costs of heavy military load carriage (6–9), particularly at slower walking speeds (2), steep downhill grades (10), and while carrying contemporary military equipment (7). Recently, two new equations were developed to better predict body mass–specific energy expenditure during standing and level walking (2) as well as walking on uphill and downhill grades (10). However, these newer equations were developed using data collected without heavy external loading and must be adapted to account for modern military equipment.

Predicting energy expenditure during heavy load carriage is complicated. Walking energy expenditure rises nonlinearly with increased load relative to body mass (11,12). Energy costs differ depending on whether loads are carried closer or further away from the body’s center of mass (13). In addition, certain military equipment further increase energy costs by impairing walking biomechanics such as the effect of rifle carriage on arm swing and upper body coordination (14). New predictive equations that account for the metabolic costs of different types of military equipment and their load mass relative to an individual’s body mass are needed.

We sought to develop and validate an acceptably accurate equation for estimating metabolic rate (M˙) during heavy military load carriage. Our study focused on backpack loads which often impose the largest burden on dismounted warfighters (15). Ultimately, our intent is to provide mission planners, exercise scientists, and tactical strength and conditioning coaches with an enhanced metabolic cost model applicable for both military and general purposes.

## METHODS

### Design

Developing and validating an acceptably accurate equation for predicting metabolic rates during heavy military load carriage involved completing the following objectives. First, we collected steady-state metabolic rates from healthy, active military-age men and women while unloaded (0% body mass) and when carrying three backpack loads (22%, 44%, and 66% body mass) at walking speeds up to 1.97 m·s^−1^. These levels of loading were chosen to incorporate an unloaded walking condition and three equidistant backpack loads. The 44% and 66% body mass conditions correspond with the relative masses of clothing and individual equipment listed for approach and emergency approach loads, respectively, in the most recent US Army field manual for foot marches (16). In addition, backpack masses were determined relative to body mass to enable our model to be applicable for heavier absolute loading and warfighters of varying sizes. Participants carried loads in the Modular Lightweight Load-Carrying Equipment 4000 (MOLLE 4000): the US Army’s most recently developed military rucksack. Next, we developed a new model, the LCDA backpacking equation, to predict the collected metabolic rates based on characteristics of the individual, load, and walking speed. Subsequently, we conducted a *k*-fold cross-validation for an initial examination of the generalizability of the LCDA backpacking equation. Lastly, we validated the LCDA backpacking equation against seven historical reference data sets (13,17–22).

### Participants

Thirty healthy, active military-age adults (3 women, 27 men; age, 25 ± 7 yr; height, 1.74 ± 0.07 m; body mass, 77 ± 15 kg; body fat, 21.4% ± 5.0% body mass) participated in this investigation. The study sample included 28 active-duty soldiers (2 women, 26 men) and 2 civilians (1 woman, 1 man). To meet study inclusion criteria, participants were required to perform resistance or aerobic exercise for 30 min or more at least 2 d·wk^−1^, be experienced with carrying loads greater than 30% of their body mass, and be able to walk at 1.34 m·s^−1^ while carrying a backpack equal to 66% of their body mass. Participants were briefed on the purpose of the study and potential risks before giving their voluntary informed, written consent. This study was approved by the Institutional Review Board at the US Army Medical Research and Development Command (Fort Detrick, MD). Investigators adhered to Department of Defense Instruction 3216.02 and 32 CFR 219 on the use of volunteers in research.

### Procedures

Our protocol consisted of five laboratory visits, each interspaced by at least 2 rest days, which were completed by participants over a period of 20 ± 5 d. The first two visits comprised mainly of familiarization and baseline tests, the third visit involved an incremental walking test with each load, whereas the fourth and fifth visits included discontinuous standing and walking tests with different levels of loading. Before each visit, participants were instructed to avoid alcohol (>24 h), vigorous exercise (>24 h), and high-intensity exercise (>48 h), as well as caffeine, nicotine, and food intake (>10 h). Participants drank ≥500 mL water the night before and the morning of each visit to ensure they were not dehydrated. Hydration status was subsequently confirmed by checking that urine specific gravity was ≤1.030. Participants began testing at the same designated time each visit (0600 to 0900 h) wearing combat boots and standard physical training clothing (i.e., shorts, T-shirt, socks). Combat boots were worn to be consistent with footwear worn during US Army ruck marches and dismounted movements.

Participants completed incremental walking tests on a motorized treadmill (Trackmaster® TMX428; Full Vision, Inc., Newton, KS) while unloaded on the first and second visits: one while wearing running shoes and the other while wearing combat boots. The order of footwear was randomized, and the data from the combat boot trials were ultimately used for model development and validation. Participants underwent dual-energy x-ray absorptiometry (GE Lunar iDXA; GE Healthcare Lunar, Madison, WI) scans on the second visit for body composition assessment. On the third visit, each participant completed incremental walking tests in combat boots while carrying the 22%, 44%, and 66% body mass loads in a randomized order. For each incremental walking test, participants began by walking for 3 min at 1.16 m·s^−1^ and 0% grade. The treadmill speed was then increased by 0.09 m·s^−1^ every 2 min thereafter. Testing was terminated when the participant walked for 2 min at 1.97 m·s^−1^, reached a respiratory exchange ratio (RER) > 1, or was unable to sustain the treadmill speed without jogging, hopping, or running. Participants were provided 12-min rest intervals between tests.

Participants were randomly assigned to two of the four load conditions on the fourth visit and the remaining two conditions on the fifth visit. For each load, participants completed five 6-min discontinuous trials including one standing and four walking trials while wearing combat boots. Each participant walked at three fixed speeds (0.45, 0.89, and 1.34 m·s^−1^) plus the highest speed that individual walked during the incremental test with that load while maintaining a RER <1. Participants were allotted 2-min rest between speeds and 12-min rest between load conditions.

The 22%, 44%, and 66% body mass loads were carried in MOLLE 4000 backpacks. Each MOLLE 4000 was packed with rubber bumper plates, smaller ankle weights, and hard foam arranged so that the heaviest mass was closest to the body. The mass of the MOLLE 4000 itself was included in the calculated mass of each load. Participants were familiarized to the loaded conditions at the end of the first visit by completing consecutive 2-min walks at 0.45, 0.89, and 1.34 m·s^−1^ with each load.

Participants wore a two-way breathing mask (2700 series; Hans Rudolph Inc., Shawnee, KS) connected to a laboratory metabolic measurement system (TrueOne 2400; ParvoMedics, Sandy, UT) that measured oxygen uptake (V˙O_2_) and carbon dioxide production (V˙CO_2_). The metabolic measurement system was allowed to warm up for >60 min and a minimum of two flowmeter and gas analyzer calibrations before testing in accordance with manufacturer instructions.

Although steady-state aerobic metabolism can be reached within 1–2 min (23), we implemented additional controls to ensure valid measurements of V˙CO_2_ and V˙O_2_. The initial walking speed of the incremental test was set at 1.16 m·s^−1^ for a modest rest-to-work transition followed by minimal speed increases (0.09 m·s^−1^) to elicit a gradual rise in V˙CO_2_ and V˙O_2_. In this way, additional time was allotted to reach steady-state M˙ at higher walking speeds (e.g., metabolic rates were measured during the 5th minute of the incremental test when walking at 1.25 m·s^−1^ vs the 21st minute when walking at 1.97 m·s^−1^). We checked that the coefficient of variation for both V˙CO_2_ and V˙O_2_ was less than 10% (24) between the first and second 30 s of the final minute of each trial. The RER for each measurement was required to be within the respiratory quotient range of 0.7 to 1. If a participant’s RER surpassed 1, the incremental test was terminated and the data from that speed were excluded from analysis. The final speed for the load-matched discontinuous test was then adjusted to 0.09 m·s^−1^ below the highest speed completed during the incremental test. As final checks, we quantified the agreement between M˙ measured during first and second 30 s of the final minute of each trial as well as M˙ measured during the incremental and discontinuous tests at matched loads and speeds.

Steady-state measurements were averaged over the final minute of each speed. Metabolic rates were calculated via indirect calorimetry using the equation from Kipp et al. (25) derived from the updated nonprotein respiratory quotient table of Péronnet and Massicotte (26). Resting metabolic rates were estimated using the Cunningham equation (27) based on lean body mass measured by dual-energy x-ray absorptiometry. For historical reference data sets that only reported V˙O_2_, an RER of 0.85 was assumed when calculating M˙.

### Model development

As in our previous work (2), we quantified the effect of walking speed (*S*; m·s^−1^) on M˙ using a constant term (*a*), fractional exponent term (*bS*^1/*c*^), and a quartic term (*dS*^4^). The nonexercise component of the overall M˙ is accounted for by the inclusion of the individual’s resting metabolic rate (*M˙*_Rest_). External load has a multiplicative, nonlinear effect on walking M˙ with light loads causing small increases and heavier loads incurring disproportionately greater costs (4,7,11). Consequently, we modeled the effect of backpack load (*L*_Bp_; backpack mass divided by body mass) using a power function (*xL*_Bp_*^y^*). The overall equation is written as follows:


$$\overset{M\cdot }{}\;\left(W\cdot {\mathrm{kg}}^{-1}\right)={\overset{M\cdot }{}}_{\text{Rest}}+\left(a+bS^{1/c}+dS^{4}\right)\left(1+{\mathrm{xL}}_{\text{Bp}}{}^{y}\right)$$ [1]

### *k*-Fold cross-validation

We conducted a *k*-fold cross-validation (10,28) to evaluate the generalizability of the LCDA backpacking equation. First, we assigned each of the three female participants to a different group and evenly split the 27 male participants between the three groups in a random manner. Next, we fit a model to the data from two of the groups with the remaining holdout group used as a test data set to evaluate the model. This process was repeated until each group was used as the test data set once.

### External validation

We externally validated the LCDA backpacking equation on data collected in 81 healthy US Army soldiers and civilians (17 women, 64 men) from seven historical reference studies (13,17–22). Each study involved respiratory gas exchange measurement for indirect calorimetry during steady-state treadmill walking with backpacks in temperate laboratory conditions. All data from each of the seven studies were analyzed excluding trials that involved alternative modes of exercise (19,20,29), uphill or downhill slope walking (22), or abnormal load distributions (13). Table 1 summarizes the participants and conditions from each study. Participants were between 18 and 41 yr, 1.57 and 1.90 m tall, and 48 and 120 kg in weight. Treadmill speeds ranged between 1.12 and 1.79 m·s^−1^ with backpack loads reaching up to 62% body mass.

**TABLE 1:** Participant characteristics (mean ± SD) and range of conditions (minimum, maximum) from reference studies.

Study	*n* (F)	Age (yr)	Height (m)	Body Mass (kg)	Pack	Method	Time (min)	Speed (m·s^−1^)	Load (%)
Daniels et al. (17)	10 (0)	24 ± 3	1.72 ± 0.04	67 ± 8	PB	TS	—	1.56	0, 41
Daniels et al. (18)	4 (0)	22 ± 1	1.77 ± 0.03	71 ± 1	PB, SP, TX, Z2	TS	18	1.12, 1.56	0, 26
Harman et al. (19)	14 (0)	24 ± 4	1.73 ± 0.09	82 ± 17	AL, CM	SM	6	1.34, 1.79	0, 62
Harman et al. (20)	14 (0)	28 ± 7	1.75 ± 0.05	81 ± 9	NS	SM	6	1.56	0, 21
Kirk and Schneider (21)	11 (11)	22 ± 4	1.66 ± 0.03	58 ± 8	AL, FP	SM	15	1.43	33
Obusek et al. (13)	12 (0)	—	1.73 ± 0.09	79 ± 13	AL, CM	SM	6	1.56	0, 52
Santee et al. (22)	16 (6)	23 ± 5	1.77 ± 0.07	76 ± 15	AL	SM	20	1.34	3, 36

AL, All-Purpose Lightweight Individual Carrying Equipment (ALICE); CM, commercial backpack; F, number of female study participants; FP, field pack large with internal frame; NS, nonspecific backpack; *n*, number of total study participants; PB, packboard; SP, standard combat pack; SM; SensorMedics 2900 Metabolic Cart; TX, T53-8 experimental combat pack; TS, Tissot spirometer; Z2, experimental UK Z-2 combat pack.

### Statistical analyses

Data are displayed as mean ± SD unless stated otherwise and were analyzed using R (version 4.0.3; R Foundation for Statistical Computing, Vienna, Austria) (30). The α level for statistical significance was set to 0.05. Mixed-effects models were fitted by restricted maximum likelihood and included random effects of participant on intercepts to account for participant-specific effects. We calculated 90% confidence interval (CI) using the bootstrap percentile method (31). Trial data were visually inspected for erroneous data points, and outliers were additionally screened using the median absolute deviation from the median approach recommended by Leys et al. (32). Metabolic rate estimates were compared with estimates from two models developed for military load carriage, the Minimum Mechanics model (33) and the equation from Pandolf et al. (4).

Coefficients for the LCDA backpacking equation were fit using nonlinear mixed-effects models via the “nlme” package (34). We first analyzed paired differences with linear mixed effects models via the “lme4” package (35) to calculate a properly weighted bias estimate by accounting for the dependence among repeated measures as well as potentially unequal number of data points between participants. We then conducted equivalence testing using the two one-sided *t*-test and tested if the 90% CI of the mean paired difference was within equivalence limits equal to 10% of the mean measured M˙ (36). Accuracy and level of agreement were evaluated by the bias and concordance correlation coefficient (CCC), respectively.

## RESULTS

The final speed determined for the discontinuous trials during the load-matched incremental tests decreased with backpack load relative to body mass (0%, 1.96 ± 0.04 m·s^−1^; 22%, 1.86 ± 0.12 m·s^−1^; 44%, 1.67 ± 0.16 m·s^−1^; 66%, 1.48 ± 0.12 m·s^−1^). Metabolic rates measured during first and second 30 s of the final minute of each trial were similar (bias, −0.08 ± 0.29 W·kg^−1^; 90% CI, −0.06 to −0.10 W·kg^−1^; CCC, 0.989). Likewise, we found strong agreement between M˙ measured during the incremental and discontinuous tests at matched loads and speeds (bias, −0.02 ± 0.43 W·kg^−1^; 90% CI, −0.09 to 0.05 W·kg^−1^; CCC, 0.962).

Across all trials, the mean measured walking M˙ was 6.12 ± 2.14 W·kg^−1^. Metabolic rate estimates were statistically equivalent to the measured M˙ in each *k*-fold cross-validation step (*P* < 0.01) as the 90% confidence limits of the mean paired difference were within 10% of the mean walking M˙ (*k* = 1 [−0.45 to −0.07 W·kg^−1^]; *k* = 2 [−0.14 to 0.34 W·kg^−1^]; *k* = 3 [−0.11 to 0.36 W·kg^−1^). Overall, M˙ estimates from the LCDA backpacking equation were accurate and precise (bias, −0.01 ± 0.63 W·kg^−1^; 90% CI, −0.15 to 0.11 W·kg^−1^; CCC, 0.964). The final LCDA backpacking equation for calculating M˙ based on resting M˙, walking speed (*S*; m·s^−1^), and backpack load (*L*_Bp_; backpack load divided by body mass) fit to the combined data set was as follows:


$$\overset{M\cdot }{}\;\left(W\cdot {\mathrm{kg}}^{-1}\right)={\overset{M\cdot }{}}_{\text{Rest}}+\left(0.19+1.78S^{0.58}+0.27S^{4}\right)\left(1+1.96{L_{\mathrm{Bp}}}^{1.36}\right)$$ [2]

**FIGURE 1:**
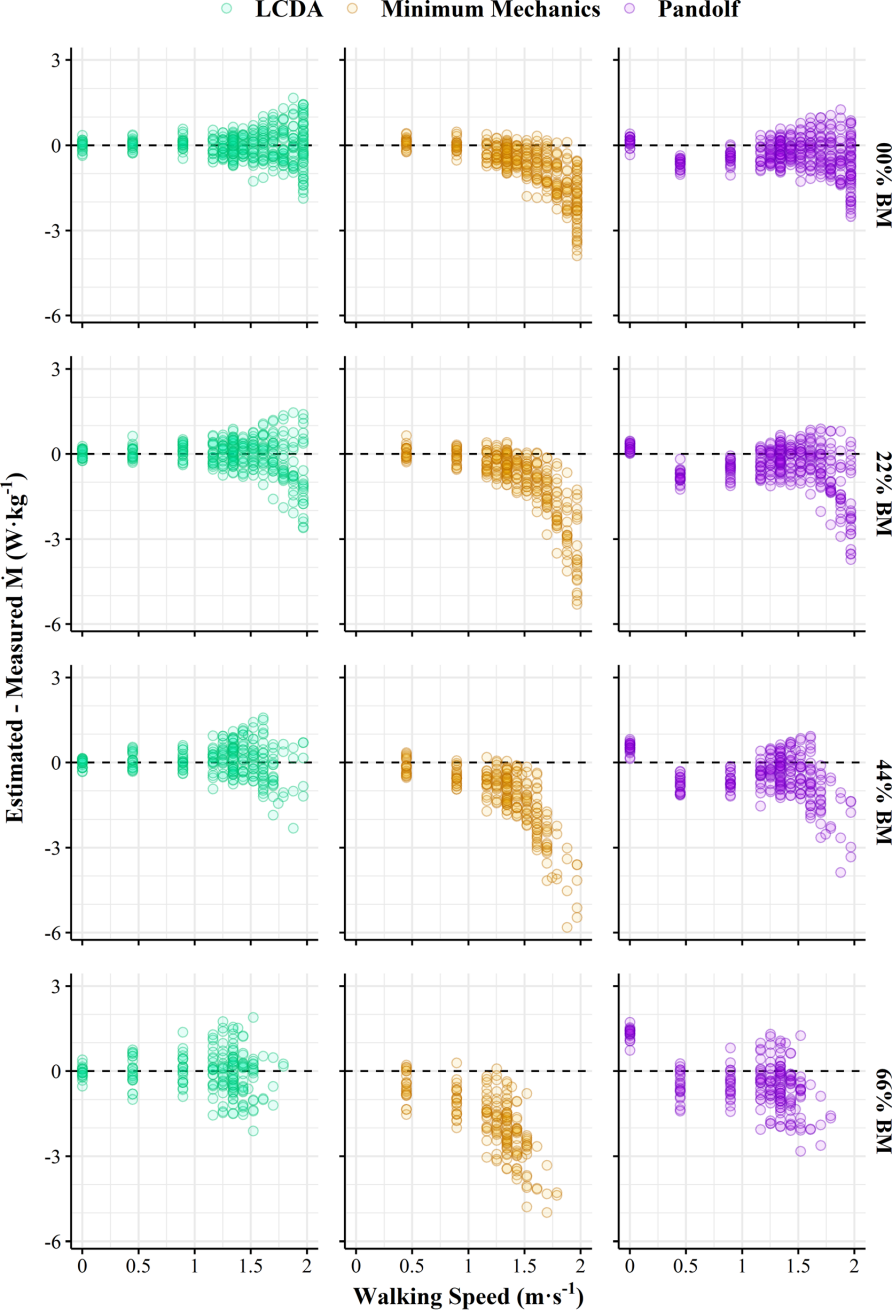
Paired differences between estimated and measured metabolic rates (M˙) across walking speed by backpack load (0%, 22%, 44%, and 66% body mass) from study trial data. Dashed line, line of zero difference.

Figure 1 shows paired differences between estimated and measured M˙ across walking speed by backpack load from the final equation and the two reference equations. The LCDA backpacking equation more accurately and precisely predicted walking M˙ (bias, 0.01 ± 0.63 W·kg^−1^; 90% CI, −0.13 to 0.15 W·kg^−1^; CCC, 0.952) than the Minimum Mechanics model (bias, −1.06 ± 1.12 W·kg^−1^; 90% CI, −1.23 to −0.91 W·kg^−1^; CCC, 0.664) and Pandolf equation (bias, −0.44 ± 0.74 W·kg^−1^; 90% CI, −0.59 to −0.30 W·kg^−1^; CCC, 0.908).

**FIGURE 2:**
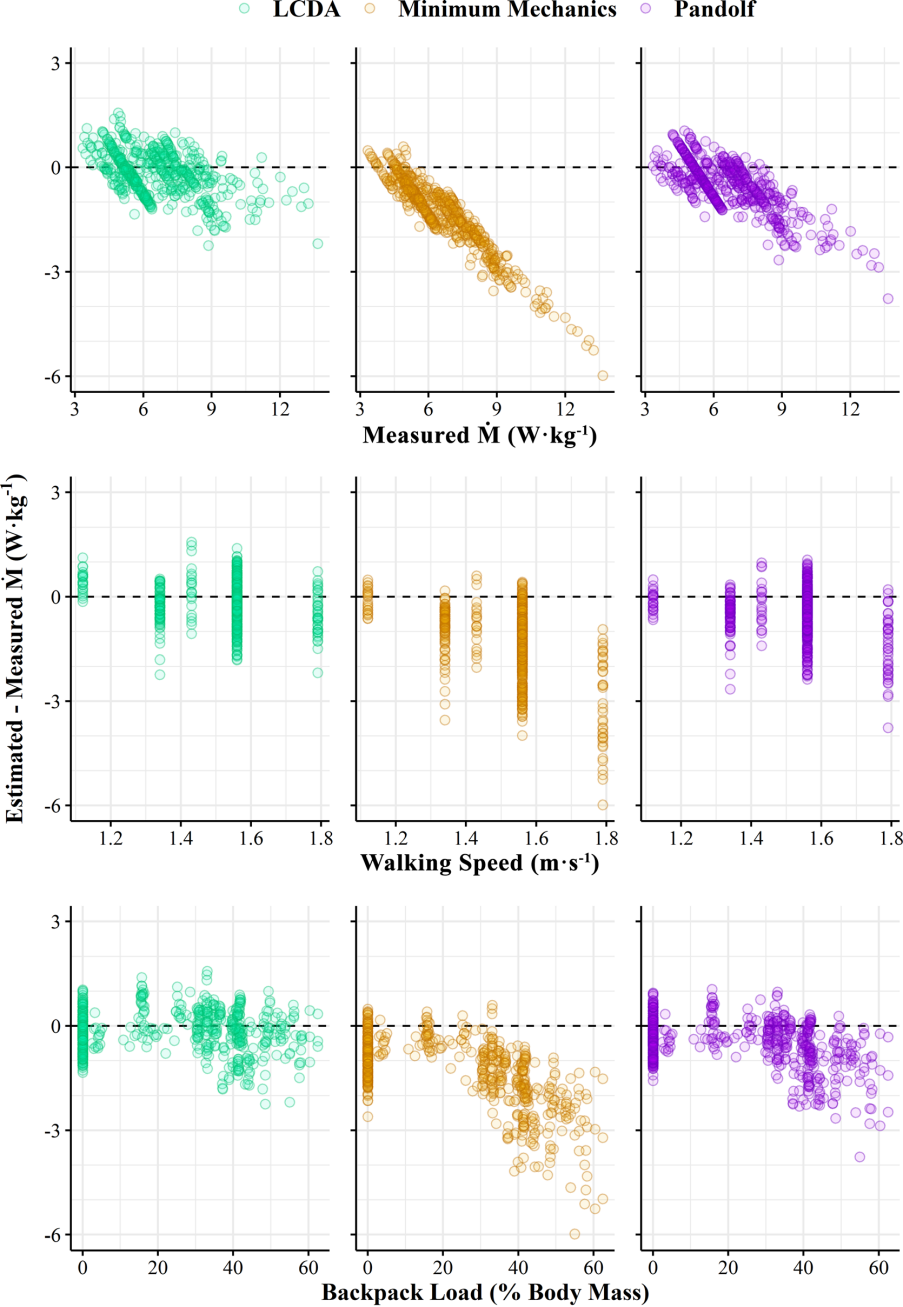
Paired differences between estimated and measured metabolic rates (M˙) across measured M˙, walking speed, and backpack load from historical reference study data (13,17–22). Dashed line, line of zero difference.

Figure 2 displays paired differences between model estimates and the M˙ measurements from the reference studies over measured M˙ (Fig. 2A), backpack load (Fig. 2B), and treadmill speed (Fig. 2C). Estimates from the LCDA backpacking equation were more accurate and precise (bias, −0.08 ± 0.59 W·kg^−1^; 90% CI, −0.18 to 0.01 W·kg^−1^; CCC, 0.926) than estimates from the Minimum Mechanics model (bias, −1.23 ± 1.02 W·kg^−1^; 90% CI, −1.37 to −1.09 W·kg^−1^; CCC, 0.513) and Pandolf equation (bias, −0.46 ± 0.67 W·kg^−1^; 90% CI, −0.56 to −0.37 W·kg^−1^; CCC, 0.863). In addition, the 90% confidence limits around the mean paired difference were within 10% of the mean (6.50 W·kg^−1^), indicating statistical equivalence (*P* < 0.01).

## DISCUSSION

Our study demonstrates that the LCDA backpacking equation is an accurate estimator of the metabolic costs of carrying modern military rucksacks loads up to 66% body mass at walking speeds up to 1.97 m·s^−1^. The new equation met our predetermined criterion for statistical equivalence during each round of *k*-fold cross-validation and when externally validated against data from seven historical reference studies. When compared with two existing predictive models, the LCDA backpacking equation showed greater accuracy for predicting M˙ during standing with loads and slow to fast speed walking. Future research can expand upon the present study by quantifying the metabolic costs of walking with other types of loads carried by dismounted warfighters on various types of surfaces and slopes.

The LCDA backpacking equation contains several distinguishing features that allow improved accuracy for a wide variety of conditions. Primarily, the LCDA backpacking equation incorporates the intercept, fractional exponent speed term, and quartic speed term previously identified (2) as necessary for estimating M˙ during standing as well as walking at various speeds. The backpack load component (1.96*L*_Bp_^1.36^) accounts for the nonlinear effect of modern military rucksack loads weighing up to 66% body mass. The modular structure of the LCDA backpacking equation enables compatibility with previously developed terrain coefficients (*η*) (37,38) and the LCDA-graded walking equation (10). For example, the LCDA-graded walking equation (*M˙*_Grade_; equation 3), modified for use with decimal grades (*G*; rise/run) for consistency with the load component, can be readily incorporated into the new LCDA backpacking equation (equation 4):


$${\overset{M\cdot }{}}_{\text{Grade}}\;\left(W\cdot {\mathrm{kg}}^{-1}\right)=\;34\mathrm{SG}\left(1-1.05^{1-1.1^{100G+32}}\right)$$ [3]

$$\overset{M\cdot }{}\;\left(W\cdot {\mathrm{kg}}^{-1}\right)={\overset{M\cdot }{}}_{\text{Rest}}+\left[0.19+\eta \left(1.78S^{0.58}+0.27S^{4}+{\overset{M\cdot }{}}_{\text{Grade}}\right)\right]\left(1+1.96{L_{\mathrm{Bp}}}^{1.36}\right)$$ [4]

The current study meticulously evaluated the quantitative relationship between load carried in a modern military rucksack and metabolic energy expenditure while walking at various speeds. The derived equation herein provides users with accurate estimates of the metabolic costs specific to backpack loads. Prior studies have demonstrated that the Pandolf equation, which was developed to predict metabolic costs of carrying backpack loads (4), is less accurate when accounting for loads added by explosive ordnance disposal suits (8), weighted vests (33), or combinations of combat equipment (e.g., torso body armor, chest/hip webbing, assault rifle) (7,9). As evident in Figures 1 and 2, the assumption that body mass and external load mass cause the same linear increase in metabolic energy expenditure, which is the basis of the Minimum Mechanics model (33), is not true for military load carriage. Schertzer and Riemer (39) identified the requirement for separate equations to predict M˙ when walking with loads positioned on either the back, knee, or ankle. These findings emphasize the need for a model that can account for the metabolic costs of carrying various types of military equipment individually or in combination.

Precise estimation of the metabolic costs of walking at the highest speeds remains an area for improvement for existing load carriage models that include body mass as the only physiological input (Figs. 1, 2). Although height has been used in the past when predicting walking energy expenditure (40), a larger study sample would be required to quantify these relationships across the diverse spectrum of body shapes and sizes that encompass healthy military populations (41). Body mass and foot contact time have been previously demonstrated to predict ambulatory activity energy expenditure across a wide range of species (42). Height potentially introduces mass, lean mass, center of mass, and gait variables that may affect movement efficiency in humans. Even larger carried weights (>75% of body mass) may force a change in gait, with a nonlinear threshold transition in energy costs (43). The recent introduction of female warfighters into combat positions (44) makes the need for models that can accurately predict M˙ for both men and women more urgent. However, this objective necessitates a more extensive and dedicated research project than the current investigation, as prior studies on sex differences in the metabolic costs of load carriage have shown conflicting results (45,46).

The influence of body composition on the metabolic costs of military load carriage is another essential topic for future research. Our study only accounted for the relationship between lean body mass and resting M˙ (27). Notably, Silder et al. (47) found that normalizing the metabolic cost of walking per kilogram lean body mass reduced sex differences during unloaded and loaded trials. Studies comparing the net metabolic costs of walking of obese adults versus normal-weight counterparts are in disagreement (48–50). In our data set, we found a positive association between percent body fat (%BF) and overestimation of M˙ (paired difference, −0.153 + 0.009%BF), although the slope coefficient was not significant (*P* = 0.60). Explicating the relationship between body composition and net M˙ during military load carriage requires additional data to achieve necessary statistical power.

Importantly, a number of factors may elevate the metabolic costs of military load carriage over time beyond steady-state estimates. Oxygen uptake exceeds steady-state projections toward maximal values during severe-intensity exercise when fatigue and the progressive loss of muscular efficiency lead to the development of the V˙O_2_ slow component (51). Metabolic heat production increases with prolonged cold or heat stress exposure because of adaptations related to thermogenesis and evaporative heat loss respectively (52). Oxygen uptake drift may occur because of shifts in substrate utilization rather than changes in the energy cost of the activity (53). Accounting for all of these factors would require additional inputs for physical fitness and fatigue, integration of a thermophysiological model, and dietary intake and energy availability information. Our study minimized the influence of drift on steady-state measurements with frequent rest intervals, hydration checks, thermoneutral environmental conditions, and an aerobic exercise intensity limit (RER = 1). Ultimately, the steady-state M˙ estimates provided by the LCDA backpacking equation are a useful foundation for more comprehensive physiological models with time-varying components.

## CONCLUSIONS

The newly derived LCDA backpacking equation provides close estimates of steady-state metabolic energy expenditure when carrying heavy military backpack loads. This novel formula enables improved M˙ predictions across a wide range of walking speeds and external loads carried in both modern and historical military equipment. These advances allow end users to further optimize thermal-work strain monitoring, sports nutrition, and hydration strategies.
